# Gender differences in myocardial salvage and clinical outcome in patients with acute reperfused ST-elevation myocardial infarction

**DOI:** 10.1186/1532-429X-13-S1-O86

**Published:** 2011-02-02

**Authors:** Ingo Eitel, Steffen Desch, Georg Fuernau, Matthias Gutberlet, Gerhard Schuler, Holger Thiele

**Affiliations:** 1 Heart Center Leipzig, Leipzig, Germany

## Introduction

There is conflicting evidence regarding gender-based differences in outcomes in patients after myocardial infarction. Recently, a study demonstrated greater myocardial salvage assessed with SPECT imaging in women than in men. However, SPECT imaging has several limitations including its low spatial resolution particularly of subendocardial infarcts and as a result cardiac magnetic resonance imaging (CMR) has emerged as the reference method to assess myocardial salvage.

## Purpose

The aim of this study was to investigate whether there are gender-associated differences in the amount of myocardial salvage assessed by CMR and clinical outcome in ST-elevation myocardial infarction (STEMI) patients reperfused by primary percutaneous coronary intervention (PCI).

## Methods

In this study we included 87 women (mean age 63±12 years) and 262 men (mean age 69±13 years) with STEMI undergoing primary PCI <12 hours after symptom onset. The primary end point was myocardial salvage (area at risk minus infarct size) assessed by T2-weighted and contrast-enhanced CMR. The secondary end point was the occurrence of major adverse cardiovascular events (MACE) defined as death, reinfarction and occurrence of new congestive heart failure within 6 months after the index event.

## Results

The amount of myocardium at risk (35.3±13.5%LV versus 34.6±14.0%LV, p=0.68) and final infarct size (16.7±13.1%LV versus 17.9±12.4%LV) did not differ significantly between women and men. Consequently, the myocardial salvage index was similar between groups (53.7±26.2 versus 49.9±26.4, p=0.25). After adjustment for baseline characteristics, female gender was no independent predictor of greater myocardial salvage after PCI.

Regarding clinical outcomes there was a trend of an increased MACE rate in women (21% versus 15%, p=0.09) as well as a significantly increased mortality rate in female myocardial infarction patients (14% versus 8%, p=0.03).

## Conclusions

The efficacy of primary PCI (myocardial salvage) in patients with STEMI is not gender-dependent. However, female STEMI patients have worse clinical outcomes than men, irrespective of the salvaged myocardium at risk.

